# Overcoming Heterogeneity of Antigen Expression for Effective CAR T Cell Targeting of Cancers

**DOI:** 10.3390/cancers12051075

**Published:** 2020-04-26

**Authors:** Sareetha Kailayangiri, Bianca Altvater, Malena Wiebel, Silke Jamitzky, Claudia Rossig

**Affiliations:** Department of Pediatric Hematology and Oncology, University Children´s Hospital Muenster, 48149 Münster, Germany

**Keywords:** Cellular immunotherapy, CAR T cells, gene engineering, tumor-associated antigens, tumor microenvironment

## Abstract

Chimeric antigen receptor (CAR) gene-modified T cells (CAR T cells) can eradicate B cell malignancies via recognition of surface-expressed B lineage antigens. Antigen escape remains a major mechanism of relapse and is a key barrier for expanding the use of CAR T cells towards solid cancers with their more diverse surface antigen repertoires. In this review we discuss strategies by which cancers become amenable to effective CAR T cell therapy despite heterogeneous phenotypes. Pharmaceutical approaches have been reported that selectively upregulate individual target antigens on the cancer cell surface to sensitize antigen-negative subclones for recognition by CARs. In addition, advanced T cell engineering strategies now enable CAR T cells to interact with more than a single antigen simultaneously. Still, the choice of adequate targets reliably and selectively expressed on the cell surface of tumor cells but not normal cells, ideally by driving tumor growth, is limited, and even dual or triple antigen targeting is unlikely to cure most solid tumors. Innovative receptor designs and combination strategies now aim to recruit bystander cells and alternative cytolytic mechanisms that broaden the activity of CAR-engineered T cells beyond CAR antigen-dependent tumor cell recognition.

## 1. Introduction

### 1.1. Chimeric Antigen Receptor (CAR) Targeting of Cancer 

The cellular immune system has emerged as a highly active treatment modality against cancer. Antibody inhibitors of immune checkpoints can invigorate T cells with native specificity for tumor-associated neoantigens, which are present in the tumor microenvironment (TME) of some cancers, to induce and maintain tumor regression [1,2]. However, many tumors, especially those with a low tumor mutational burden, lack spontaneous T cell infiltration and activation and continue to be ignored by the cellular immune system despite checkpoint inhibition [3,4,5]. In the absence of preexisting adaptive immunity, adoptive transfer of tumor-antigen specific T cells can be an effective tool to establish therapeutic antitumor immune responses. Antitumor T cells can be generated either by transfer of high-avidity T cell receptor (TCR) genes into polyclonal T cells to recognize HLA (human leukocyte antigen)-restricted tumor-associated peptides [6] or by T cell engineering to express chimeric antigen receptors (CARs) [7]. CARs are synthetic receptors that recognize cancer cells via surface antigens independent of peptide presentation to the TCR. Antigen-binding domains, usually derived from monoclonal antibodies, are artificially linked to T-cell activating intracellular signaling components. CARs are expressed in T cells by gene transfer technologies [8,9]. Upon antigen engagement, they induce downstream signaling and T cell activation responses that result in target cytolysis, cytokine release and antigen-dependent T cell proliferation. Following a first generation of CARs solely relying on either Fc receptor endodomains or the TCRζ chain for intracellular signaling [7], a second generation was developed by adding costimulatory signaling domains derived from either CD28 [10] or the tumor necrosis family member 4-1BB [8]. Integrated costimulation enables CAR T cells to proliferate and expand in response to interaction with target antigens and has proven to be a key prerequisite for complete and durable clinical responses to CAR T cell therapy [11]. For the use in humans, CAR T cells are manufactured from a lymphocyte apheresis product, followed by adoptive transfer to the patient after a cycle of preparative chemotherapy, usually with fludarabine and cyclophosphamide, to optimize conditions for antigen-driven in vivo expansion [12]. The most extensively developed CAR T cell products to date are directed against the B lineage antigen CD (cluster of differentiation) 19. They have been found to induce complete remissions in 60 to 93% of patients with chemorefractory precursor B cell acute lymphoblastic leukemias (ALL) [11,12,13,14,15] and 50 to 75% responses among patients with B cell non-Hodgkin lymphomas (NHL) [16,17], leading to marketing authorization for two CAR T cell products since 2017. Axicabtagene ciloleucel is a product containing CD28 costimulation and is approved for the treatment of adult patients with large B cell lymphomas after failure of conventional therapy. Tisagenlecleucel, a product with costimulation derived from 4-1BB, has marketing authorization for the same indication and in addition for pediatric and young adult patients with relapsed and refractory CD19-positive ALL. Typical acute toxicities of CD19-specific CAR T cell therapy are fever and hypotension caused by systemic release of inflammatory cytokines (CRS, cytokine release syndrome) and encephalopathy-like neurotoxicities [18]. CAR T cells containing costimulatory domains derived from 4-1BB can functionally persist in vivo as memory populations for prolonged periods, often years, and effectively prevent disease relapse [11]. This creates a plateau of relapse-free survival in patients with previously incurable cancers and supports the development of CD19 CAR T cells as stand-alone cancer therapeutics, to increase the probabilities of survival and to replace more toxic components of current regimens. 

A major and still unachieved goal in the field is to extend the potential of CAR T cell therapy also to solid tumors.

### 1.2. Antigen Prerequisites for CAR T Cell Targeting

Safe and effective clearance of cancer cells by CAR T cells requires both selective and reliable expression of the target antigen on the cell surface. The most advanced CAR T cell products are all directed against B lineage antigens and thus to hematological cancers. Besides B lymphocyte antigen CD19 [11,16,17], these include CD22 in ALL [19], CD20 in NHL [20], B cell maturation antigen (BCMA) in multiple myeloma [21], and CD30 in Hodgkin lymphoma [22]. B-cell associated antigens are exceptional targets, since their coexpression is restricted to normal B cells and/or their progenitors. By on-target toxicity, B-lineage directed CAR T cells cause B cell aplasia with subsequent hypogammaglobulinemia in all patients in which the T cells persist [12]. Although this is a relevant late effect of therapy, it is manageable by immunoglobulin replacement therapy. Thus, even though surface expression of B-lineage antigens is not a tumor-specific feature, the fact that humans can live without B cells allows the safe use of B-cell targeted therapeutics. Identifying antigens exclusively expressed on tumor cells and not on indispensable normal cells is a key challenge for extending safe and effective CAR T cell therapy to solid cancers. Antigens targeted by CAR T cells in first clinical studies in solid tumors include disialoganglioside G_D2_ [23,24,25,26], human epidermal growth factor receptor 2 (HER2) [27], epidermal growth factor receptor variant III (EGFRvIII) [28,29], carcinoembryonic antigen (CEA) [30], interleukin (IL)13Rα2 [31], prostate-specific membrane antigen (PSMA) [32], neural cell adhesion molecule L1 (NCAM-L1, CD171) [33], receptor tyrosine kinase like orphan receptor 1 (ROR1) [34], and B7H3 [35]. Even though none of these antigens is truly tumor-specific, no clinical on-target toxicities attributable to crossreactivities with normal cells were observed. However, at the same time, CAR T cell therapy failed to induce complete and durable remissions in any of these studies, raising the concern that the high safety profile may be attributed to low in vivo activity of CAR T cells and that acute toxicities could still emerge with the use of the same antigen and more effective CAR designs. 

CAR T cell-mediated clearance of cells requires antigen expression above a detection threshold that varies with individual antigens and CARs. In B cell precursor ALL and in B cell NHL, CD19 is usually homogeneously expressed at high densities on all cells derived from the malignant clone which can be explained by an essential role of CD19 in B lineage development and a functional contribution to disease maintenance [36,37]. Although CAR T cell recognition of CD19 is characterized by a very low detection threshold, requiring less than 100 antigens per cell [38], CD19-negative subpopulations can escape recognition, grow out, and produce relapses of the disease [11,13,19,39,40,41]. Antigen escape is typically caused by acquired somatic mutations in the CD19 gene which results in a loss of membrane anchorage of the extracellular domain while preserving expression of cytosolic components of CD19 with their functional roles [39,42]. A less frequent type of antigen-negative escape is a switch of the malignant phenotype from an undifferentiated, myeloid precursor clone [43]. Thus, even for the treatment of B-lineage malignancies with CD19-specific CAR T cells, heterogeneity of antigen expression remains a serious obstacle.

In solid cancers, the prerequisites for effective antigen-based clearance of disease are substantially less favorable than for CD19-targeted leukemia and lymphoma therapy. Due to a lack of cancer-driving antigens selectively expressed on the tumor cell surface, most of the antigens used are not critical for tumor growth and maintenance, thus low-expression variants do not have a clonal disadvantage. Moreover, solid cancers often have diverse cellular phenotypes and individual target antigens are not expressed on all tumor cell subpopulations, fundamentally challenging the concept. 

Indeed, in preclinical models of pancreatic and prostate cancer, CAR T cells directed against mucin-1 (MUC1) and prostate stem cell antigen (PSCA) were unable to eradicate solid tumors, and tumor escape was attributed to tumor cells expressing low densities of target antigen [44]. In a first-in-human pilot study of CAR T cells targeting EGFRvIII in recurrent glioblastoma, post-therapeutic biopsies contained reduced levels of EGFRvIII antigen expression compared to pre-therapeutic tumors in five of seven patients [28]. Failure of CAR T cells to detect and eliminate these antigen-low tumor cells may have contributed to treatment failure in this study.

With the exception of CD19 [38], CAR-mediated T cell recognition typically requires high levels of target expression to fully activate the T cell. Even CAR T cells against the alternative B-lineage antigens CD20 [45], CD30 [46], and CD22 [19,47] in B cell malignancies need high amounts of surface antigen for effective clearance of the malignant cells. In clinical studies using CD22-redirected CAR T cells to treat ALL, leukemia escape was caused by emergence of leukemia variants with low-level CD22 expression in the absence of mutations, as observed with CD19 [19]. Low densities of surface CD22 were further associated with poor early expansion and short-term activity of CAR T cells as well as with impaired ability to persist as memory populations [47]. 

An example in a solid tumor that illustrates the need for high-level antigen expression on the targeted tumor cells is the anaplastic lymphoma kinase (ALK), which is overexpressed on the cell surface in neuroblastoma [48]. ALK-specific CAR T cells had limited efficacy against neuroblastoma cells, and this was attributed to insufficient ALK target density on neuroblastoma cells to trigger optimal CAR T function [49]. 

Our group uses the disialoganglioside antigen G_D2_ to direct CAR T cells against solid cancers of childhood. As a marker of immature neuroectodermal cells, G_D2_ is abundantly surface-expressed in neuroblastoma which originates from undifferentiated neuroectoderm [50,51]. Pediatric bone sarcomas, osteosarcoma, and Ewing sarcoma can also express G_D2_ on the cell surface [52,53], however, only a proportion of these cancers express the antigen and expression levels often vary within individual tumors both at primary diagnosis and at relapse [54]. G_D2_-low or G_D2_-negative subpopulations in sarcomas will primarily resist targeting with G_D2_-specific CAR T cells. 

Overall, density of antigen expression can affect all components of an antitumor CAR T cell response. The selection of tumor variants lacking adequate antigen expression to trigger optimal CAR T cell activation remains a challenge for the effective clinical use of CAR T cell therapeutics. 

## 2. Pharmaceutical Strategies for Upregulating CAR Target Antigens in Cancer Cells

Several strategies and agents have demonstrated their ability to sensitize cells of both hematological and solid cancers to CAR T cell targeting by increasing surface expression of individual antigens above the detection threshold of CARs (Table 1). 

**Figure 1:**
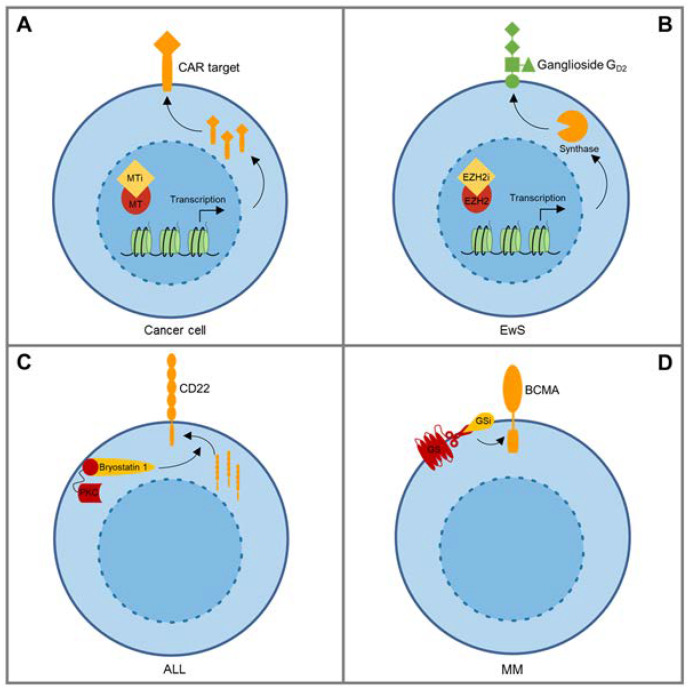
Pharmaceutical agents upregulate CAR target antigen expression in cancer cells by various mechanisms. (**A**) Methyltransferase inhibitors (decitabine, azacytidine) can increase surface expression of chimeric antigen receptor (CAR) targets such as MUC1 in cancer cells [44]. (**B**) EZH2 inhibiting agents induce gene expression of enzymes involved in G_D2_ synthesis and upregulate G_D2_ surface expression in GD2-low or G_D2_-negative Ewing sarcoma cells [56]. (**C**) Bryostatin1, a protein kinase C (PKC) modulating agent, increases CD22 surface expression by an unresolved mechanism proposed to involve changes in membrane trafficking [47]. (**D**) Small-molecule γ-secretase (GS) inhibitors (GSI) prevent cleavage of the CAR target BCMA from the surface of multiple myeloma cells [57].

One of these approaches is based on the use of epigenetic modulators, such as the DNA methyltransferase inhibitors azacytidine and decitabine, which can increase expression of tumor-associated intracellular antigens for classical presentation to T cells via TCRs [58,59]. Anurathapan et al. were the first to provide preclinical evidence that epigenetic antigen modulation can sensitize tumor cells to CAR-mediated recognition [44]. They found that decitabine exposure upregulates MUC1 on the surface of pancreatic cancer cells, effectively sensitizing tumor cells to in vitro cytolysis by MUC1-specific CAR T cells (Figure 1A). A more recent example for the use of epigenetic agents as sensitizers for CAR T cell therapy is upregulation of ligands of natural killer (NK)G2D by valproic acid, a potent histone deacetylase (HDAC) inhibitor [55]. NKG2D is an activating receptor expressed on the cell surface of NK cells. Ligands such as the structural major histocompatibility complex class I homologs (MIC)A and MICB are widely surface-expressed in both solid and hematological cancers and therefore attractive therapeutic targets. Most CARs against NKG2D ligands are composed of the full-length native NKG2D receptor, coupled to costimulatory and TCRζ domains [60,61]. In a first-in-human clinical trial in patients with acute myeloid leukemia (AML), NKG2D ligand-redirected CAR T cells had limited efficacy, which was attributed to insufficient levels of ligand expression on the surface of leukemic blasts of the patients [62]. The same group now found that NKG2D ligand expression on AML blasts can be selectively enhanced by preincubation with valproic acid, resulting in augmented antileukemic activity in functional in vitro assays [55].

While carbohydrate antigens such as ganglioside G_D2_ cannot be directly regulated by agents modulating gene expression, expression of the enzymes that mediate stage- and context-dependent biosynthesis of gangliosides during organ development underlie epigenetic modulation [63], providing a rationale for the use of epigenetic agents to upregulate G_D2_ in cancer cells. In neuroblastoma cells, which typically express G_D2_ at uniform high levels, the HDAC inhibitor vorinostat was indeed found to further increase G_D2_ expression in vitro, and this was associated with induction of a critical enzyme in G_D2_ biosynthesis, GD2 synthase (GD2S) [64]. To upregulate G_D2_ to homogenous expression levels in Ewing sarcoma we chose an alternative epigenetic inhibitor, against Enhancer of Zeste Homolog 2 (EZH2) (Figure 1B). EZH2 in Ewing sarcoma is a downstream target of the product of the disease-defining translocation, Ewing sarcoma breakpoint region 1-Friend leukemia integration 1 transcription factor (EWSR1-FLI1) [65] and an important contributor to self-renewal, tumorigenicity and phenotypic heterogeneity in this cancer [65,66,67]. In preclinical studies, inhibitors of EZH2 reliably, reversibly, and selectively upregulated G_D2_ surface expression in G_D2_-low or GD2-negative Ewing sarcoma cells to levels inducing effective antigen-specific activation of CAR T cells [56]. G_D2_ upregulation by EZH2 inhibition was associated with the known enzymatic function of EZH2, removal of methylation marks at histone H3K27, and with upregulated gene expression of GD2S as well as the upstream enzyme, GD3 synthase (GD3S) [56]. 

Thus, administration of epigenetic agents could be an attractive strategy to prevent immune escape of tumor cells that express target antigens below the detection thresholds of CAR T cells. Clinical trials of such combinations should employ a window design with the use of the upregulating agent, followed by re-biopsies to confirm the postulated effects on antigen expression levels and their tumor selectivity in human patients.

Besides epigenetic modulators, alternative antigen upregulating strategies have been reported. Ramakrishna et al. have used a protein kinase C modulator, bryostatin 1, to upregulate CD22 antigen on CD22-low ALL cells [47] (Figure 1C). Single-dose exposure increased CD22 expression in vitro and enhanced the antitumor activity of CD22-redirected CAR T cells in a preclinical xenograft model [47]. The underlying mechanism was found to involve posttranslational improvement of membrane trafficking of CD22, though details remain unresolved. Thus, combining CD22 CAR T cell therapy with preceding administration of bryostatin could allow to eliminate even low CD22-expressing leukemic cell subpopulations.

Another example is the capacity of small-molecule γ-secretase inhibitors to increase expression of BCMA on multiple myeloma cells by preventing cleavage of the antigen from the cell surface [57] (Figure 1D). Besides increasing target antigen density, γ-secretase inhibitors reduce levels of soluble BCMA in serum which interfere with CAR T cell recognition of malignant plasma cells. Indeed, loss of BCMA and variable expression levels of the antigen at different disease sites were found to contribute to mixed responses and relapse in patients treated with BCMA-specific CAR T cells [68]. In preclinical xenograft models, γ-secretase inhibitors improved recognition of cancer cells by BCMA-specific CAR T cells, reduced soluble BCMA levels and enhanced antitumor activity [57]. Short term treatment of patients increased not only expression levels of BCMA on antigen-expressing myeloma cells, but also the percentage of BCMA-positive tumor cells. A clinical trial investigating the feasibility, safety, and efficacy of the combination of BCMA-specific CAR T cell therapy with repeated doses of small-molecule γ-secretase inhibitors is ongoing (NCT03502577).

The design of combination therapies with pharmacological upregulators should consider effects of the agents on tumor cells, CAR T cells, and components of the TME. A critical concern is the tumor selectivity of antigen expression which must be preserved after systemic administration of upregulating agents to avoid off-target toxicities. In addition, manipulation of antigen expression in cancer cells by epigenetic modulators could affect their malignant phenotype and even promote aggressive, tumor-propagating, or metastatic behavior. Ideally, the agent would have antitumor activity in itself while supporting the in vivo function and persistence of T cells and dampening regulatory mechanisms that limit T cell trafficking and action in the TME. Epigenetic reprogramming indeed is a key mechanism of memory T cell formation, homeostasis, and the plasticity of recall responses [69]. The effects of individual epigenetic agents on T cell subpopulations and tumor-infiltrating bystander cells are unpredictable and will have to be addressed. 

## 3. CAR T Cell Engineering for Combinatorial Targeting of Two or More Antigens

Selective pharmaceutical upregulation of targets to homogeneous levels will be applicable only for a minority of cancer-associated surface antigens. As CAR T cells against single antigens will be insufficient for durable long-term antitumor responses in most hematological and solid cancers, strategies are being developed that allow cotargeting of more than a single antigen, thereby extending the activity of CAR T cells to several phenotypic subpopulations of the disease (Table 2). Preclinical efforts, now followed by clinical studies, have been focusing on B cell malignancies, specifically the combinations of the B-lineage antigens CD19 and CD22 in ALL [70] and CD19 and CD20 in NHL [71,72].

**Figure 2:**
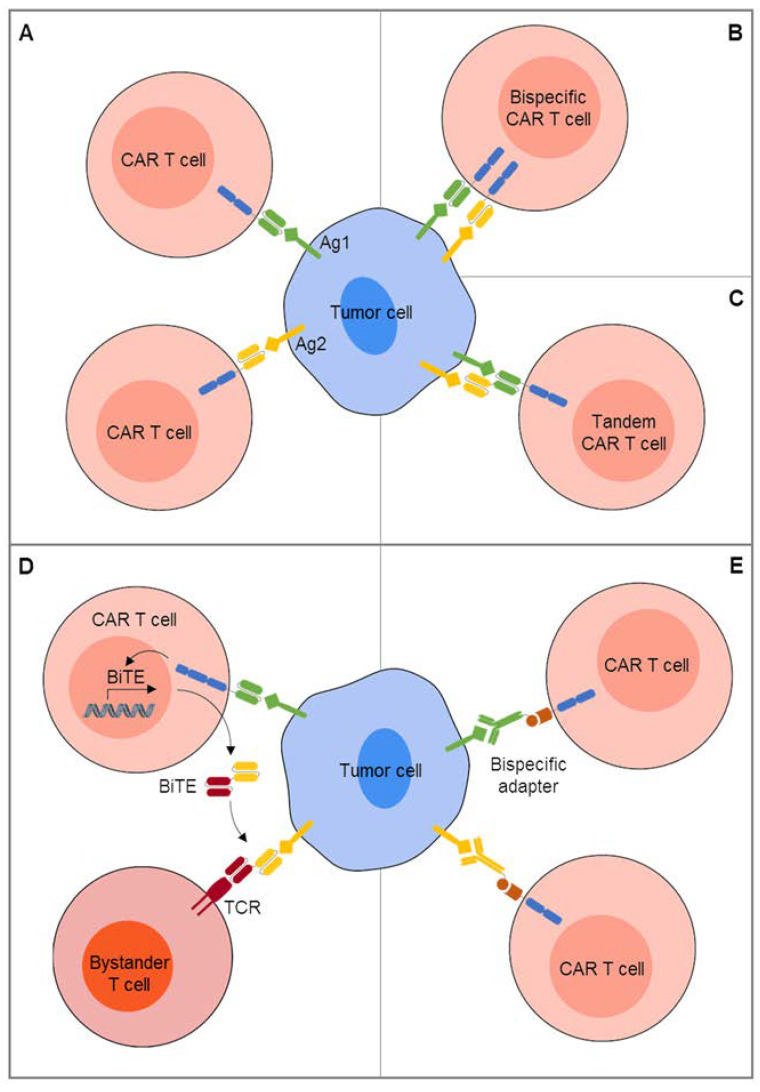
Combinatorial CAR targeting of two or more antigens. (**A**) Two CAR T cell products with individual single antigen specificities are administered simultaneously or sequentially. (**B**) Two CARs with individual single antigen specificities are coexpressed in one T cell. (**C**) Antibody-derived recognition domains for two individual antigens are combined in a single CAR to coengage both antigens. (**D**) CAR T cells are engineered to release a bispecific T cell engager (BiTE) to recruit bystander T cells against a second tumor-associated surface antigen. (**E**) T cells are engineered to express universal CARs with specificity for a molecule not expressed on the surface of any human cells, then bispecific adapters combining this molecule with antibody fragments against individual antigens are added.

Dual-redirected CAR T cell therapy can be achieved by coadministration of two pooled single-specific CAR T cell products administered together [75] (Figure 2A). In first clinical studies, two CAR T cell products with single-antigen specificities for CD19 and CD22, respectively, were given sequentially [73] or simultaneously [74]. Compared to previous reports on single-antigen targeted CAR T cell therapy [18], excessive CRS and/or neurotoxicities were not observed in either of the two studies. CD19-negative and/or CD22-low relapses still occurred in a proportion of patients, and neither of the studies was powered to compare the incidence of antigen-negative relapses following single-antigen targeting. A concern with this strategy is that the stronger product will outcompete the weaker product in the circulation, reducing the cotargeting capacity of pooled cells. Alternatives are designs that combine recognition of two antigens in one gene-modified T cell so that either antigen A or antigen B triggers T cell activation. While cotransduction of T cells with two different CAR transgenes creates three subpopulations of T cells expressing either one of the two CARs or both, bicistronic vectors that encode two different CARs allow simultaneous expression [85] (Figure 2B). An early example of CAR-mediated dual antigen targeting was CD19/CD123 bispecific T cells, designed to prevent disease relapses by CD19-negative clones detected at baseline in CD123-positive B cell precursor ALL [75]. In a mouse model mimicking CD19-negative disease relapse, CD19/CD123 dual-targeted T cells had increased antitumor activity compared to CD19-specific CAR T cells alone. A technical challenge of coexpressing two CAR transgenes in one T cell even by single vectors is inferior expression of either of the two CARs. To effectively interact with both antigens, the spacer length of the two CARs must be adjusted to overcome variable distances of the two epitopes from the cell membrane.

Novel dual-antigen targeted CAR T cells combine the antigen-recognition exodomains for two individual antigens to coengage both antigens together in a bivalent immune synapse [19,71,77,78] (Figure 2C). Such bivalent-bispecific receptors, also called tandem CARs, mediate T cell activation in response to either one of the two targets and can induce super-additive cytokine secretion upon encounters of both targets simultaneously. This approach is technically challenged by compromised protein folding that can prevent optimal epitope recognition. To overcome the complexities of single chain variable domain (scFv) folding, Ahn et al. have suggested expression of single-domain antibody mimics rather than scFvs [79]. In a preclinical proof-of-concept study, two of these molecules were effectively assembled in single gene expression cassettes, resulting in efficient targeting of two epitopes of a single target antigen and also of two distinct antigens expressed in various solid cancers, EGFR and HER2 [79]. 

To recruit bystander T cells against a second tumor-associated surface antigen, CAR T cell targeting can be combined with the release of bispecific T cell engagers (BiTEs) (Figure 2D). Iwahori et al. first described so-called engager T cells that secrete bispecific molecules linking T cells to the epithelial cancer-associated antigen EPH receptor A2 (EphA2) for bystander T cell mediated in vitro cytolysis [86]. More recently, CAR T cells directed against EGFRvIII were designed to secrete engagers against wild-type EGFR for local recruitment of bystander T cells against EGFRvIII-negative tumor cell subpopulations in glioblastoma [80] to overcome the limited clinical efficacy of EGFRvIII single-antigen targeted T cells [28]. 

Finally, modular constructs that rely on adapters for antigen recognition can extend the reactivity of T cells against tumors with diverse phenotypes [81,83,84] (Figure 2E). In this approach, T cells are engineered to express so-called universal CARs with extracellular antigen recognition domains not for a tumor-associated antigen, but a molecule not expressed on the surface of any human cells, such as biotin, fluorescein isothiocyanate (FITC), or a nuclear autoantigen. Bispecific adapters, i.e. FITC-labeled tumor specific antibodies, are added to cross-link T cells to a specific antigen while the adapter is present in the blood stream. Sequential or simultaneous infusions of adapters directed against individual antigens could allow to address a broad range of heterogenously expressed antigens. Due to the limited half-lives of the adapters, antigen targeting will be limited to periods of continuous adapter infusions, enhancing the safety of targeting each individual antigen. Clinical results of multiple antigen targeting with adapter CAR T cells have not yet been reported.

To what extent dual antigen targeting can prevent the emergence of new or preexisting antigen loss variants remains to be demonstrated even in B-lineage neoplasias, and the concept is not easily transferred to solid cancers with their high intra- and interpatient variability of surface antigen expression and lack of truly tumor-selective targets. In preclinical studies in a prostate cancer model, even combined targeting of two antigens, MUC1 and PSCA, was insufficient to eliminate the tumor [44]. Tumor cells expressing neither of the two antigens at sufficient intensities for adequate CAR T cell recognition continued to escape. Bielamowicz et al. used a trivalent T cell product coexpressing three individual CAR molecules against HER2, EphA2, and EGFRvIII by a single tricistronic transgene [87]. The three CARs were successfully expressed at comparable densities in transduced T cells. Compared to single-specific CAR T cells, trivalent CAR T cells had higher antitumor activity against patient-derived xenografts that reflected the antigen diversity of the individual tumors. 

Technically, the numbers of concomitantly expressed CARs able to interact with antigen cannot be increased indefinitely. Multiplex targeting of human cancers will require systematic screening to identify optimal antigen combinations. Since solid tumors usually share antigens with normal tissues, on-target off-tumor toxicities will remain an obstacle for the clinical development of multitargeted CAR T cells. 

## 4. Extending the Activity of CAR T Cells by Recruitment of Bystander Cells

Diverse expression of target antigens among tumor cells may not invariably lead to tumor escape if additional effector cells can be recruited and activated in order to eradicate antigen-low or -negative cells in an antigen-independent manner. In an experimental mouse model, antigen-independent, interferon (IFN)-γ mediated destruction of tumor stroma was found to contribute to effective eradication of solid tumors by CAR T cells [88]. Various strategies to broaden the CAR T cell induced immune response by cytokine modulation of the TME and recruitment or invigoration of bystander cells with antitumor effector functions have been reported. An attractive feature of these strategies is their potential to simultaneously enhance the function of the CAR T cells and enable their long-term functional persistence by creating a supportive immune-stimulatory milieu. 

**Figure 3:**
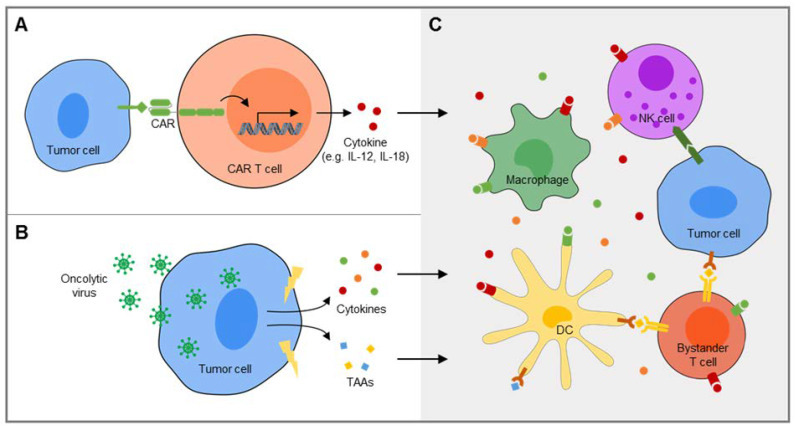
Recruitment and activation of bystander cells for amplification of CAR T cell-induced immune responses. (**A**) CAR T cells can be engineered to secrete a specific immune-stimulatory cytokine, e.g. IL-12 or IL-18, into the tumor microenvironment upon engagement of the CAR target antigen. (**B**) Oncoloytic viruses infect tumor cells and lead to the release of endogenous tumor-associated antigens along with proinflammatory danger signals. (**C**) Proinflammatory cytokines released by CAR T cells (**A**) or tumor cells following immunogenic cell death induced by oncolytic viruses (**B**) broaden and enhance the anticancer immune response by various mechanisms, including recruitment and activation of NK cells, reprogramming of immunosuppressive myeloid cells, e.g. macrophages, towards immune stimulation and presentation of tumor-associated antigens (TAAs) on dendritic cells (DCs) and other antigen-presenting cells to endogenous bystander T cells with native T cell receptors (TCRs) directed against these antigens.

One approach is to engineer CAR T cells to deliver a specific cytokine into the TME as a transgenic product (Figure 3A). The first human cytokine investigated for this purpose was IL-12, which has multiple stimulatory effects on both adaptive and innate immune effector cells, including natural killer (NK) cells and both CD8+ cytolytic and CD4+ helper T cells. Indeed, CAR T cells that release interleukin (IL)-12 were found to effectively eradicate tumors in murine models [89,90,91,92]. In a first-in-human clinical trial in patients with melanoma, the clinical impact of adoptive transfer of CAR T cells transduced to secrete IL-12 was limited by systemic toxicities, attributed to high serum levels of the cytokine [93]. Inducible secretion of the cytokine in response to interaction with the CAR target may be an effective means to avoid systemic toxicities [94,95]. More recently, interleukin (IL)-18 emerged as alternative cytokine candidate with overlapping immunological effects and better tolerance by humans [96,97,98]. In preclinical models of both B cell cancers and adenocarcinomas, IL-18-enhanced CAR T cells created a supportive TME characterized by increases of proinflammatory macrophages and activated NK cells which enhanced proliferation and cytolytic activity of CAR T cells as well as their activity in xenograft models [96,97,98]. In an immune-competent syngeneic mouse model, IL-18-secreting CART were capable of enhancing long-term survival of mice inoculated with both antigen-positive and antigen-negative pancreatic adenocarcinoma cells, and this was found to be mediated at least in part by recruitment of endogenous effector T cells [96].

Alternatively or in addition to immune-stimulatory cytokines, CAR T cells can be engineered to secrete single-chain variable domains that interrupt engagement of immune-inhibitory checkpoint receptors, such as programmed death (PD)-1 in PD-L1 positive tumors [99,100]. Checkpoint inhibitors secreted by CAR T cells can serve to enhance their own effector functions and prevent their exhaustion, and at the same time may unleash preexisting T cells with native receptor specificity for tumor-associated (neo)antigens. Smith et al. suggested the use of implantable biopolymer devices to deliver CAR T cells along with a small-molecule vaccine adjuvant directly to solid tumors, to achieve lysis of CAR antigen-positive tumor cells by high concentrations of immune cells along with a second wave of a broader immune response to tumor-associated (neo)antigens released by the tumor [101]. 

An attractive combination partner for adoptive therapy of solid cancers with CAR T cells are oncolytic viruses with their multiple functions (Figure 3B): Direct infection of tumor cells leads to immunogenic cell death [102] which could contribute to CAR T cell mediated tumor cell destruction by an alternative, antigen-independent mechanism. At the same time, proinflammatory danger signals by oncolytic viruses restore bystander immune cell functions and reverse the suppressive TME [103] (Figure 3C). In tumors with preexisting tumor-primed T cells, virus-mediated cell death may further result in the presentation of antigen to bystander T cells through their TCRs and amplify a broader host anti-tumor immune response by epitope spreading [102,104]. Oncolytic viruses can further be manipulated to deliver immunostimulatory cytokines and/or checkpoint inhibitors into the tumor which facilitate tumor infiltration with CAR T cells and improve their function, as demonstrated in preclinical studies in a variety of solid tumor models [105,106,107,108]. Vice versa, CAR T cells can be used as vehicles to deposit oncolytic viruses into the TME to enhance and extend their own activity [109]. Even non-oncolytic viruses, such as inactivated influenza used for seasonal vaccination, can augment local antitumor immune responses by stimulating tumor antigen-specific T cells along with antigen-presenting cells [110]. Thus, viral immune adjuvants could extend CAR-T cell mediated tumor destruction by a more diverse subsequent T cell memory response that includes CAR antigen-negative escape clones. 

The major limitation for a broader antitumor response of CAR T cells is that it requires the presence of preexisting antigen-specific antitumor T cells in the TME. As is apparent from the restricted activity of checkpoint inhibitors, many tumors fail to express (neo)antigens recognized by TCRs and lack infiltrating T cells in their TME [4,5]. In these tumors, including pediatric cancers and sarcomas, the limited antigen specificity of CAR T cells will be more difficult to overcome. Still, recruitment and activation of bystander cells such as NK cells and other innate immune effector cells by oncolytic viruses may contribute to CAR T cell mediated tumor cell destruction. Moreover, antigen-independent cytolysis can be induced by CAR T cells via upregulation of Fas ligand, resulting in subsequent cytolytic interactions with CAR-antigen negative, Fas-expressing tumor cells [111]. In an embryonal carcinoma model with heterogeneous CD30 antigen expression, Hong et al. found that CD30-specific CAR T cells used this mechanism to eliminate CD30-negative or dim-expressing tumor cells. To exploit the Fas/FasL pathway for broader and more effective CAR T cell targeting even beyond tumors with natural high Fas expression, the group suggested oncolytic viral delivery of Fas to Fas-negative tumor cells to sensitize tumor cells to Fas-mediated bystander lysis.

## 5. Conclusions

Lack of suitable surface antigens and heterogeneity of their expression remains a significant limitation to the widespread use of CAR T cells for immunotherapy of cancer. Pretreatment with select epigenetic modulators and other pharmaceutical agents could be effective to upregulate individual CAR targets and thereby increase the potency of CAR T cell therapy. Advanced CAR designs enable simultaneous targeting of two or even multiple antigens. Engineering or combination strategies that amplify CAR T cell-induced responses against single antigens by antigen-independent mechanisms may overcome barriers that currently limit the activity of CAR T cells in solid tumors. This includes the action of proinflammatory cytokines in the TME and the recruitment of additional effector cells directed against alternative target antigens or acting in an antigen-independent manner. Animal models are inadequate to reproduce the complex interplay between CAR T cells, antigen-positive and antigen-low/negative tumor targets and bystander immune populations in the microenvironment of human tumors. Well-designed clinical studies and immunological readouts will be needed to address the safety and therapeutic value of strategies aiming to amplify and broaden CAR-induced immune responses.

## Figures and Tables

**Table 1:** Pharmaceutical strategies (Preclinical).

Strategy	CAR Target	Entity	Ref.
Epigenetic modulation			
DNA methyltransferase inhibitor: Decitabine	MUC1	Pancreatic cancer	[44]
HDAC inhibitor: Valproic acid	NKG2D ligands	AML, T-ALL	[55]
EZH2 inhibitors: GSK126, tazemetostat	GD2	Ewing sarcoma	[56]
Posttranslational modification			
Protein kinase C modulator: Bryostatin 1	CD22	B cell precursor ALL	[47]
Preventing antigen cleavage from cell surface			
-secretase inhibitor	BCMA	Multiple myeloma	[57]

ALL, acute lymphoblastic leukemia; MUC1, mucin 1; NKG2D, natural killer G2D; AML, acute myeloid leukemia; BCMA, B cell maturation antigen.

**Table 2:** Combinatorial targeting strategies.

Clinical Studies			
Two Single-Antigen Products	CD19, CD22	B Cell Precursor ALL	[73,74]
Preclinical			
Bicistronic transgene	CD19, CD123	B cell precursor ALL	[75]
Tricistronic transgene	CD19, CD20, CD22	B cell precursor ALL	[76]
Bivalent-bispecific receptor (tandem CARs)	CD19, CD22	B cell precursor ALL	[19]
	CD19, CD20	Non Hodgkin lymphoma	[71,72]
	HER2, IL13Rα2	Glioblastoma	[19,77,78]
Bispecific single-domain antibody mimics	HER2, EGFR	Pancreatic cancer	[79]
CAR T cells secreting BiTEs	EGFR, EGFRvIII	Glioblastoma	[80]
Adapter CARs	PSMA, CA-IX, FRα, NK1R	Model tumor	[81]
	CD19, CD22	B cell precursor ALL	[82]
	Mesothelin, FRα, EpCAM	Ovarian cancer	[83]
	CD33, CD123	AML	[84]

ALL; acute lymphoblastic leukemia; HER2, human epidermal growth factor receptor 2; IL13Rα2, interleukin 13 receptor α2; EGFR, epidermal growth factor receptor; BiTE, bispecific T cell engager, EGFRvIII, epidermal growth factor receptor variant III; PSMA, prostate-specific membrane antigen; CA-IX, carbonic anhydrase IX; FRα, folate receptor α; NK1R, neurokinin 1 receptor; EpCAM, epithelial cell adhesion molecule.

## Abbreviations

**Abb.**	**Full Name**
ALK	anaplastic lymphoma kinase
ALL	acute lymphoblastic leukemia
AML	acute myeloid leukemia
BCMA	B cell maturation antigen
BiTE	Bispecific T cell engager
CA-IX	carbonic anhydrase IX
CD	cluster of differentiation
CEA	carcinoembryonic antigen
CRS	cytokine release syndrome
EGFR	epidermal growth factor receptor
EGFRvIII	epidermal growth factor receptor variant III
EpCAM	epithelial cell adhesion molecule
EphA2	EPH receptor A2
EWSR1-FLI1	Ewing sarcoma breakpoint region 1-Friend leukemia integration 1 transcription factor
EZH2	enhancer of Zeste Homolog 2
FR	folate receptor
GD2S	GD2 synthase
GD3S	GD3 synthase
HDAC	histone deacetylase
HER2	human epidermal growth factor receptor 2HLA human leukocyte antigen
IFN-	interferon-
IL13R 2	interleukin 13 receptor 2
IL-12	interleukin-12
IL-18	interleukin-18
MIC	major histocompatibility complex class I homolog
MUC1	mucin 1
NHL	non Hodgkin lymphoma
NK cell	natural killer cell
NKG2D	natural killer G2D
NK1R	neurokinin 1 receptor
PD-1	programmed death-1
PSMA	prostate-specific membrane antigen
PSCA	prostate stem cell antigen
PSMA	prostate-specific membrane antigen
ROR1	receptor tyrosine kinase like orphan receptor 1
scFv	single chain variable domain
TCR	T cell receptor
TME	tumor microenvironment
